# [Retracted] Nerve growth factor and its receptors on onset and diagnosis of ovarian cancer

**DOI:** 10.3892/ol.2025.15332

**Published:** 2025-10-13

**Authors:** Xiaolin Yu, Zhaoxia Liu, Rui Hou, Yijun Nie, Rensheng Chen

Oncol Lett 14: 2864–2868, 2017; DOI: 10.3892/ol.2017.6527

Following the publication of this paper, it was drawn to the Editor's attention by a concerned reader that, regarding the immunohistochemical data shown in Fig. 6, the TrkA and p75NTR panels appeared to show an overlap, albeit the images were stretched differently. Furthermore, in the “Patients with ovarian cancer” column, the NGF and p75NTR panels also appeared to show an overlap, again with a different stretching factor. Finally, the distribution and coloring of these samples appeared to be very different comparing between the two columns, as if different stainings may have been applied: The reader considered that the left column may have shown H&E staining experiments, whereas the right column panels appeared to have been stained with an antibody.

Upon performing an independent analysis of the data in this paper in the Editorial Office, it came to light that certain of the data in Fig. 6 were strikingly similar to data that had already been submitted for publication in an article written by different authors at different research institutes, also to the journal *Oncology Letters*.

Owing to the fact that the contentious data in the above article had already been submitted for publication prior to its submission to *Oncology Letters*, the Editor has decided that this paper should be retracted from the Journal. The authors were asked for an explanation to account for these concerns, but the Editorial Office did not receive a reply. The Editor apologizes to the readership for any inconvenience caused.

